# Fluoride intake during pregnancy: calculation of realistic exposure scenarios for individual risk assessment

**DOI:** 10.1007/s00204-025-04143-8

**Published:** 2025-08-31

**Authors:** A. Sonnenburg, M. Batke, G. Damm, H. Foth, A. Freyberger, J. G. Hengstler, A. Mangerich, F. Partosch, Th. Schupp, K-M. Wollin, J. Vom Brocke, U. Gundert-Remy

**Affiliations:** 1 Department Pesticides Safety, German Federal Institute for Risk Assessment, Berlin, Germany; 2 University of Applied Sciences Emden/Leer, Emden, Germany; 3 Department of Hepatobiliary Surgery and Visceral Transplantation, Leipzig University Medical Center, Leipzig, Germany; 4 Institute of Environmental Toxicology, University of Halle, Halle/Saale, Germany; 5 Formerly Bayer AG, Wuppertal, Germany; 6 Leibniz Research Centre for Working Environment and Human Factors, Dortmund University, Dortmund, Germany; 7 Universityof Potsdam, Department of Nutritional Toxicology, Institute of Nutritional Science, Potsdam, Germany; 8 Ramboll Deutschland GmbH, Essen, Germany; 9 University of Applied Sciences Muenster, Steinfurt, Germany; 10 Formerly Public Health Agency of Lower Saxony, Hannover, Germany; 11 European Chemicals Agency, Helsinki, Finland; 12 Charité-Universitätsmedizin Berlin, Berlin, Germany

**Keywords:** Fluoride health effects, Pregnant women, Fluoridated salt, Consumption of black/green tea, Exposure assessment

## Abstract

Using the methodological approach of systematic review several regulatory bodies and associations have performed an assessment of existing evidence on the adverse effects of fluoride. The most recent review by EFSA published in 2025 recommended a fluoride level in drinking water of below 1.5 mg/L and a maximum total daily intake of 3.3 mg of fluoride as safe level with particular respect to pregnant women and the exposed unborn child. Our work focusses on individual exposure aspects, evaluating the contribution of major sources, specifically those which can be influenced by personal behaviour. We demonstrate that black or green tea and fluoridated household salt—even at salt intake levels below the population 25th percentile—are main contributors of the daily fluoride exposure in the population. Using the safe level proposed by EFSA, we have calculated different exposure scenarios that may be used by individuals to estimate their specific exposure to fluoride from known sources. Considering that the safe level proposed by EFSA can be exceeded e.g. by drinking more than two cups (300 mL) of black or green tea daily and using even low amounts of fluoridated salt, our findings highlight the need for clear labelling of the fluoride content of products to allow informed consumer choices.

**Supplementary Information:**

The online version contains supplementary material available at 10.1007/s00204-025-04143-8.

## Introduction

Since the 1940s, fluoride has been added to drinking water to reduce dental caries which might still be a public health problem in many countries. In the 2021 assessment of the global burden of diseases, the prevalence of dental caries is reported to be 29% for permanent teeth and around 7% for primary teeth (Institute for Health Metrics and Evaluation 2025). That is, 2.2 billion people with permanent teeth and around 525 million children are affected worldwide. The World Health Organization (WHO) attributed this global public health problem to excessive intake of sugars and inadequate supply with fluoride (World Health Organization 2022).

Despite the fact that the benefit from fluoride intake on dental health has been demonstrated, possible risks need to be weighed against these benefits and this topic is continuously widely discussed. The European Food Safety Authority’s (EFSA) Scientific Committee published a document, assessing the consumer risk of fluoride in food and drinking water, including the contribution from other sources of exposure (EFSA Scientific Committee 2025). In the assessment they included animal and world-wide epidemiological studies, including those from endemic fluoride areas. The EFSA opinion proposed a safe level of 3.3 mg fluoride per day specifically for pregnant women. EFSA’s analysis suggested, with some uncertainties, that fluoride exposure in pregnancy may adversely affect the developing brain based on several studies indicating that exposure to drinking water above 1.5 mg/L is associated with lower IQ scores in children. Therefore, the most vulnerable sub-population is the foetus and the most relevant sub-population in terms of exposure considerations are pregnant women according to EFSA. The proposed value is near the level of the adequate intake of 0.05 mg/kg for adults, set by EFSA’s Panel on Nutrition, Novel Foods, and Food Allergens (NDA Panel) in 2013 which was derived based on the maximum effect on dental caries and the minimal effect on dental fluorosis in epidemiological studies (Agostoni et al. 2013).

Possible reductions of IQ levels in children from prenatal fluoride exposure are being critically discussed. Choi et al. (2012) analysed epidemiological studies, mainly from China and areas with relatively high naturally occurring fluoride concentrations in drinking water, and came to the conclusion that the data suggest a relevant IQ decrement in children. Twelve years later, Taher et al. published their systematic review on health effects of fluoride in drinking water, based on epidemiological and toxicological data (Taher et al. 2024). The authors, applying Bradford-Hill criteria for causality, stated, with some uncertainty, a link between reduction of IQ levels in children at concentrations close to those seen in North American drinking water. The NTP Board of Scientific Counsellors Working Group (Taylor et al. 2025) came to the conclusion that there is moderate evidence for a reduction of IQ levels in children, the mothers of whom were exposed to fluoride in drinking water at concentrations above 1.5 mg/L during pregnancy.

Guth et al. (2021) analysed several key reviews in this field and identified possible limitations in some studies, such as insufficient adjustment for confounders (*e.g.* other neurotoxicants), and differences in applied criteria, such as inclusion of studies from regions with high fluoride concentrations in the drinking water, that may lead to differing conclusions based on the studied population. In an earlier assessment, Guth and co-workers already evaluated the evidence for human health hazards caused by exposure to fluoride with special focus on developmental toxicity considering in vitro studies, experimental animal studies, and epidemiological studies (Guth et al. 2020). They focussed on studies conducted in non-endemic fluoride areas. The authors concluded that their evaluation did not indicate health concerns with respect to adverse developmental effects at current exposure levels in European countries.

It is not the aim of this contribution to perform another systematic review addressing the link between adverse health effects and fluoride exposure nor to repeat existing exposure assessments nor to re-evaluate the safe level proposed by EFSA. This publication is aiming to demonstrate how individual behaviours, such as the daily consumption of black or green tea, drinking water intake, and the use of fluoridated salt, can influence personal cumulative fluoride exposure. Thereby, this work focuses on individual variability and real-life scenarios to illustrate how lifestyle choices impact fluoride exposure levels. This “individualised” exposure assessment with several scenarios is different from the regulatory exposure assessments which address the exposure of a whole population, typically reported as means^1^ and 95th percentiles in mg/kg body weight per day, which, however, do not allow individuals to determine their own position within the exposure distribution of the population.

For exposure scenarios analysed here, we focussed on women of child-bearing age, as far as data for this sub-population were available, since this was the sub-population specifically addressed by EFSA when deriving their safe level. Other reference values exist, *e.g.* for dental fluorosis. In Germany, the German Nutrition Society provides different reference values based on a risk for dental fluorosis for sub-populations by age ranging from 0.25 mg/d for infants to 3.0 mg/d for female and 3.5 mg/d for male adults (DGE 2022). The values for adults are in line with the value proposed by EFSA for pregnant women. EFSA also considered the association between elevated fluoride exposure and bone effects, like bone mineralisation, increased fracture risk, and skeletal fluorosis, to be sufficiently established. Although no clear threshold could be derived from the data base, evidence from human studies suggests that these effects may occur at exposure levels of around 3 mg/d (EFSA Scientific Committee 2025). Therefore, our calculations are also relevant for other adult sub-populations and other endpoints such as dental and skeletal fluorosis.

In the following, we present several scenarios which result in different levels of exposure to fluoride by varying the extent of oral intake of contributing sources. We created an array of exposure scenarios varying the amount of fluoride consumed per day of the contributing sources, from very low to very high, whereby exposure is influenced, *e.g.* by the volume of daily water intake, daily consumption of salt, or percentage of fluoridated salt, as well as the amount and type of tea consumed per day. The resulting tables allow all consumers to estimate how their individual patterns contribute to the daily overall exposure via all currently known sources. We compare the resulting daily exposure with the proposed safe level of 3.3 mg per person per day (EFSA Scientific Committee 2025), emphasising that the safe level should not be understood as a demarcation line and not every exceedance of this level constitutes a health risk.

## Material and methods

### Main food and non-food fluoride sources considered

Food, discretionary salt intake, water, and oral hygiene products (mainly toothpaste, not mouth rinses, or mouth wash) were considered as the major regular daily exposure sources of fluoride by EFSA. In addition, tea may contain high concentrations of fluoride, dependent on its origin (EFSA Scientific Committee 2025).

In line with our aim, we calculated how fluoride exposure varies by different levels of intake of fluoride-containing items with the intention to demonstrate how modifying the personal behaviour can reduce the cumulative exposure. In the scenarios we have considered:

i) the intake of household salt (discretionary salt consumption) and the percentage of fluoridated salt in discretionary intake,
ii) intake of black or green tea using the mean concentration in tea infusions and the 95th percentile of it,
iii) intake of herbal tea using the mean concentration in tea infusions and the 95th percentile of it,
iv) the consumption of drinking water per day.

Because fluoride intake from commercially prepared food cannot be accurately individualised or modified by consumers, we used the amount per day calculated by multiplying the mean values from the EFSA opinion of the fluoride exposure by food, reported in mg/kg bw per day, by the standard body weight of 70 kg to estimate the fluoride intake by food. Since fluoridated toothpaste can be considered a standard, we have calculated all scenarios including exposure from toothpaste.

### Tea

Black and green tea may contain high concentrations of fluoride dependent on its origin. Therefore, it was considered as one of the main fluoride intake sources. Whereas EFSA used the fluoride concentration in dry material in its calculations and applied a dilution factor, we selected data on fluoride concentration in infusions of tea sold in European countries, as reported in the literature (Chan et al. 2013; Jakubczyk et al. 2022; Krishnankutty et al. 2022; Malinowska et al. 2008; Pavlovič et al. 2023; Ruxton and Bond 2015; Szmagara et al. 2022; Waugh et al. 2016).

Literature values for fluoride concentration in infusions prepared with black or green tea, which was bought in European countries, varied from 0.34 to 11.12 mg/L (N = 195) with a mean of 3.13 mg/L. The fluoride concentration in infusions prepared with herbal tea varied from 0.038 to 0.077 mg/L (N = 9), with a mean of 0.056 mg/L (supplemental Table 1).

We identified data on the volume of tea consumed per day by pregnant Danish women (Krishnankutty et al. 2022) and for the general population in UK (Ruxton and Bond 2015). Values ranged between 250 mL daily and 250 mL monthly for tea drinkers in Denmark and 480 mL daily (median) up to 1230 mL daily (75th percentile) in the UK. For our scenarios, we used the following drinking volumes per day: 75 mL (0.5 cup), 150 mL (1 cup^2^), 300 mL (2 cups), 600 mL (4 cups), and 1200 mL (8 cups). Since we used the fluoride concentration in prepared infusions, it was not necessary to account for the fluoride concentration in the water, used to prepare the infusion. Hence, we used the mean value of fluoride concentration in black or green tea infusions and multiplied it with the volumes of 75 mL, 150 mL, 300 mL, 600 mL, and 1200 mL to calculate the fluoride intake by black or green tea.

### Salt

The consumption of salt in the female European population in child bearing age was estimated from data of an EU survey (European Commission 2013) that reported a range of 5.4–12.3 g per day for adult women in Europe. The most recent and detailed information was found for Germany in the DEGS1 survey (Klenow and Mensink 2016). Data on sodium intake obtained in this survey were converted to a daily salt intake by Johner et al. (2015). For women of childbearing age (18–49 years), we calculated the 25th percentile, median, and 75th percentile from this information as 6.4, 8.9, and 11.8 g per day and used these levels for our scenarios. These values are consistent with data reported for different European countries by the European Commission on their Knowledge Gateway on dietary salt and sodium (European Commission 2023).

The scenarios also took into account the percentage of the salt intake as discretionary salt (fluoridated or not). Bhat et al. (2020) reported that the discretionary salt intake worldwide varies between 5 and 86% of the total salt intake. According the EFSA NDA Panel opinion on dietary reference values for fluoride the daily amount of fluoridated discretionary salt intake is 3 g in France and 2 g in Germany (Agostoni et al. 2013), which would be 36% and 24%, respectively, of the average salt intake for women in Germany as reported by the German Federal Ministry of Food and Agriculture (BMEL 2024). In the NDA Panel opinion on dietary reference values for sodium, discretionary sodium chloride intake was reported to vary between 10% of the total salt intake in Denmark and > 33% in Italy (Turck et al. 2019). For our calculations, we used 5%, 25%, and 50% as low, medium, and high levels of discretionary salt intake, respectively, to account for the variance in reported values.

A further factor relevant for exposure was the percentage of the discretionary salt consumed as fluoridated salt for which few recent data are available in the literature. According to the French food safety authority, in 2003 around 35% of the salt in France was fluoridated (Afssa 2003). For Germany, an estimated 68% of salt sold was fluoridated in 2010 as published by the German Working Group for Dental Health (DAZ 2010). We used values of 0, 50, and 100% to characterise consumers, who (i) do not to use fluoridated salt at all, (ii) use fluoridated salt at home and non-fluoridated salt in restaurants and canteens, and (iii) use only fluoridated salt.

In several European countries,^3^ fluoride is allowed to be added to household salt for the private use, mostly between 200 and 300 mg fluoride/kg salt. A currently widely used household salt in Germany contains 310 mg fluoride/kg salt (DGE 2024). German legislation prohibits the use of fluoridated salt in commercially sold food, as well as in community catering and school meals, unless an exemption permit has been granted. Whereas in the EFSA opinion the maximal concentration of fluoride in household salt was reported to range between 200 and 250 mg/kg, and 250 mg/kg was used for the calculations, we used the higher concentration of 310 mg/kg which represents the upper limit for fluoride enrichment of salt in Germany since 2014 (IfK 2021; GMBl 2014).

### Water

For water intake, we analysed data which were reported for various European countries as presented in the EFSA opinion. The reported volumes for all beverages varied between 917 mL per day and 1895 mL per day. For the analysed scenarios we used intake volumes of 1000, 1500, and 2000 mL per day. The EFSA opinion contained data on the fluoride concentration in the basic scenario for unbottled, bottled, and tap water with upper bound values of 209, 238, and 231 µg/L, respectively. We used the median value of 231 µg/L. According to EFSA, > 86% of the drinking water concentrations of fluoride in Europe were below 0.3 µg/L and > 97% were below 0.7 mg/L.

Natural mineral water may also contribute to fluoride exposure. The legal limit value for bottled water is set at 5 mg fluoride/L by Directive 2003/40/EC (European Commission 2003). We have not considered natural mineral water in our exposure scenarios due to the lack of available data on its consumption.

### Toothpaste

Concerning toothpaste, we used the mean intake value of 0.3 mg flouride per day as calculated by EFSA for our exposure estimates. This corresponds to 9.1% of the proposed safe level, while the 95th percentile accounts for 13.6% (EFSA Scientific Committee 2025). The calculation by EFSA was based on toothpaste with the highest allowed concentration of 1500 mg F/kg toothpaste used in the European Union. In the calculated scenarios, the exposure from toothpaste was always included using the above value. However, for individuals using lower concentrated fluoridated toothpaste or fluoridated toothpaste only once daily or no fluoridated toothpaste at all, the supplemental Excel provides the possibility to select a lower value for this exposure source for individual calculations. We did not include the exposure from mouth wash or mouth rinse because of the lack of data on daily use and experimental data on the amount of fluid that is typically swallowed. However, the volume can be assumed to be low (less than 1 mL per day).

### Food

Concerning food intake, EFSA has calculated fluoride exposure as minimum and maximum of the lower, middle, and upper bound of the mean and 95th percentile. The minimum mean fluoride exposure by food in adults was calculated to be 0.47 mg per day (lower bound), 0.48 mg per day (middle bound) and 0.5 mg per day (upper bound). The respective values for the maximum were 1.14, 1.17, and 1.19 mg per day. For the 95th percentile the minimum values were 0.73, 0.74, and 0.75 mg per day (lower, middle, and upper bound, respectively) and the maximum values were 2.22, 2.26, and 2.71 mg per day (lower, middle, and upper bound, respectively). For our scenarios we selected the maximum of the middle bound of the mean exposure of 1.17 mg per day as the median value of the three maximum values.

### Calculations for all sources

The calculation of the exposure to fluoride was performed first by investigating the most influential contributors to the total exposure separately, *i.e.* without other contributors, for (i) drinking black or green tea, (ii) discretionary salt intake as fluoridated salt, (iii) water; for all three we varied the amount of intake within the indicated range. Then we calculated the intake by all contributors:

(i) salt (three different intake levels of total salt, *i.e.* the 25th percentile, median, and 75th percentile of salt intake for the population in childbearing age, three levels of discretionary salt intake of 5, 25, and 50% of the total salt intake, with two different percentages of fluoridated salt, *i.e.* 50% and 100%),
(ii) tea (0.5 cup, 1 cup, 2 cups of black or green tea),
(iii) water (intake of 1, 1.5, or 2 L/d).

In contrast to population-based exposure estimates that result in distributions of exposure with percentiles and confidence intervals for pre-defined groups of the population, we calculated exposure point estimates for different individual scenarios. We selected plausible values for the exposure by sources which can be influenced by individual behaviour. The scenarios were then calculated by simple addition of selected values per contributor permutating point estimates for intake levels as given in the list above. The selected fluoride intakes from food (1.17 mg) and toothpaste (0.3 mg) were kept constant and added to the resulting total exposure estimate in every scenario. This produced a total of 162 specific exposure scenarios (per person per day). We provide an Excel-based tool to enable individuals to calculate their personal exposure to fluoride depending on their respective consumption behaviour (cf. Supplemental Excel file). We compared exposures derived for every scenario with the safe level proposed by EFSA for pregnant women of 3.3 mg/d, expressing the exposures as mg per day and not as mg per kg body weight per day. Table 1 summarises the applied values.

**Table 1:** Main contributors to fluoride exposure, values used in exposure scenarios

Source	Consumption	Value	Unit	Reference and remarks
Food		1.17	mg/d	(EFSA Scientific Committee 2025)
Toothpaste		0.3	mg/d	(EFSA Scientific Committee 2025), value derived for swallowed toothpaste (0.2 g) with a fluoride concentration of 1500 mg/kg
Water	Water	0.231	mg/L	(EFSA Scientific Committee 2025) Median value for bottled und tap water
		1	L/d	(EFSA Scientific Committee 2025) Selected from the overview of different European countries
		1.5		
		2		
Fluoridated salt		0.31	mg/g	(GMBl 2014)
	Total salt		g/d	(Klenow and Mensink 2016; Johner et al. 2015) Total salt for women of childbearing age (18–49 years)
	25th percentile	6.4		
	Median	8.9		
	75th percentile	11.8		
	Discretionary salt	5	%	(Bhat et al. 2020) Percent of total salt intake
		25		
		50		
	Fluoridated salt	0	%	Percent of discretionary salt intake for three scenarios: i) no fluoridated salt intake at all, ii) fluoridated salt at home and non-fluoridated outside home, iii) only fluoridated salt
		50		
		100		
Black/green tea		3.13	mg/L	See Table 1 of the supplemental material
Herbal tea		0.056	mg/L	
	Tea		mL	(Krishnankutty et al. 2022; Ruxton and Bond 2015; ISO 3103:2019)
	0.5 cup	75		
	1 cup	150		
	2 cups	300		
	4 cups	600		
	8 cups	1200		

## Results

### Single contributors

#### Drinking black or green tea

Drinking black or green tea may result in fluoride exposure between 0.24 mg/d and 3.76 mg/d, based on a mean infusion concentration of 3.13 mg/L. The variation depends on the volume of tea consumed per day which can range between 0.075 L (half a cup) and 1.2 L (8 cups). This means that by black or green tea consumption in volumes of 8 cups per day the safe level would already be exceeded (Table 2). For the cumulative scenarios we restricted the black or green tea intake to 0.5 cup, 1 cup, and 2 cups.

**Table 2:** Minimum, median, and maximum fluoride contribution to the daily intake from single sources. Only the maximum value for black and green tea (corresponding to a daily consumption of 1.2 L of black or green tea) exceeds the save level of 3.3 mg/d (bold). Individual values for all scenarios can be calculated using the calculator tab in the supplemental Excel file

Single contributor	Minimum contribution [mg/d]	Median contribution [mg/d]	Maximum contribution [mg/d]
Black/green tea	0.24	0.94	**3.76**
Herbal tea	0.004	0.017	0.067
Fluoridated Salt	0.05	0.48	1.83
Water	0.231	0.347	0.462

The exposure to fluoride due to herbal tea is low. Even intake of a high volume would not noticeably contribute to the combined exposure (Table 2). Therefore, herbal tea intake was not considered in the subsequent exposure scenarios.

### Discretionary fluoridated salt intake

Calculation of the fluoride intake by discretionary salt intake was performed by varying the amount of daily salt intake levels (25th percentile, median, 75th percentile—representing low, medium, and high salt intake, respectively), the percentage of discretionary salt intake (5%, 25%, 50%) and the percentage of fluoridated salt used (50% and 100%). Eighteen results were obtained which varied between 0.05 mg/d and 1.83 mg/d. Hence, none of the intake scenarios of fluoridated salt without other contributors exceeded the safe level of 3.3 mg/d (Table 2).

### 1.3. Water intake

Data on the fluoride concentration in water in Europe indicate low concentrations in unbottled, bottled, and drinking (tap) water (0.209, 0.238 and 0.231 mg/L, respectively). We used the concentration of drinking water of 0.231 mg/L as median. When considering fluoride intake from water alone, varying daily consumptions between 1 L, 1.5 L, and 2 L results in a maximum intake level of 0.462 mg/d, which does not exceed the safe level (Table 2).

### 2. Cumulative exposure

#### 2.1. Black or green tea intake, discretionary salt intake, and water intake

Drinking black or green tea, using fluoridated salt for discretionary purposes, and the volume of water intake are all contributors that depend on personal behaviour and can influence the total level of fluoride exposure.

The cumulative fluoride exposure remains below the safe level for almost all scenarios in which the total salt intake is 6.4 g/d and the intake of black or green tea is not more than one cup. One exception is the scenario in which the discretionary salt intake is 50%, the percentage fluoridated salt is 100%, and the water intake is 2 L/d. For the scenarios with low salt intake and the consumption of two cups of black or green tea, fluoride levels exceed the safe level with an additional water intake of 2 L/d and/or high percentages of fluoridated salt (Table 2 A in the supplemental Excel file).

When the total salt intake is 8.9 g/d and the discretionary salt intake is 50% of which the percentage fluoridated salt is 100% the resulting fluoride intake exceeds the safe level of 3.3 mg/d for all volumes of consumed tea and all levels of water intake. The safe level is also exceeded when the consumption of black or green tea is two cups per day and the total salt intake is 8.9 g/d in all scenarios with a discretionary salt intake of 25% of which 100% is fluoridated as well as for 50% discretionary salt intake of which 50% is fluoridated (Table 2B in the supplemental Excel file).

When the total salt intake is 11.8 g total salt/d and black or green tea consumption does not exceed one cup, the scenarios with 50% discretionary salt intake of which 100% are fluoridated salt still result in fluoride exposure levels exceeding the safe level of 3.3 mg/d, regardless of water intake level. In addition, also in scenarios with 11.8 g total salt/d intake combined with the consumption of black or green tea of two cups with discretionary salt intake of 25% of which is 100% fluoridated salt as well as discretionary salt intake of 50% of which is 50% fluoridated salt exceed the safe level, regardless of water intake levels (Table 2 C in the supplemental Excel file).

#### 2.2. Cumulative exposure leaving out varying contributors.

##### 2.2.1. Non-consumers of black or green tea (Scenarios excluding black and green tea).

In these scenarios, total salt intake, discretionary salt intake, the percentage of fluoridated salt, and water intake were varied to calculate the cumulative fluoride exposure without consumption of black or green tea. The cumulative fluoride exposure is below the safe level for all scenarios in which the total salt intake is 6.4 g/d (Table 3 A in the supplemental Excel file) and for all scenarios in which the total salt intake is 8.9 g/d (Table 3B in the supplemental Excel file). In the scenario in which the water intake is 2 L/d and the discretionary salt intake is 50% of which 100% is fluoridated salt the safe level is reached. The cumulative fluoride exposure is also below the safe level for all scenarios when the intake of the total salt intake is 11.8 g/d unless the discretionary salt intake is 50% of which 100% is fluoridated salt regardless of water intake levels (Table 3 C in the supplemental Excel file).

##### 2.2.2. Scenarios excluding fluoridated salt

For this scenario the volume of black or green tea and the water intake were varied to calculate the cumulative exposure in the absence of fluoridated salt intake. The cumulative fluoride exposure is below the safe level for all scenarios in which the tea intake is half a cup, one or two cups of black or green tea daily (Table 4 in the supplemental Excel file). As has been pointed out, the scenario of drinking black or green tea alone in amounts of eight cups per day (*i.e.* even without any additional water) already exceeds the safe level (Table 2). Any consumption of black or green tea of more than three cups per day leads to an exceedance of the safe level, even without intake of fluoridated salt, regardless of the volume of additionally consumed water.

##### 2.2.3. Scenarios excluding both, fluoridated salt and drinking black or green tea

This scenario describes the exposure without the two main contributors for the fluoride intake, fluoridated salt and black or green tea. In this scenario only the volume of drinking water is varied (1 L, 1.5 L, and 2 L) and fluoride intake via food and toothpaste remains constant. The cumulative fluoride exposure is 1.7 mg/d, 1.83 mg/d, and 1.93 mg/d and therefore below the safe level for all scenarios of daily water intake.

As a summary, Table 3 presents some selected scenarios in which the cumulative fluoride exposure is either below, at, or above the safe level when choosing the highest possible consumption for any given contributor for the first and second, and the lowest consumption of any given contributor for the last case. In these scenarios, exposure from food and toothpaste was also included. Any other scenarios can be calculated using the calculator tab in the supplemental Excel file.

**Table 3:** Selected scenarios leading to a cumulative fluoride intake below, at, or above the safe level of 3.3 mg/d

Contributors	Below safe level	At safe level	Above safe level
Salt + water	High salt intake, 25% discretionary salt, 100% fluoridated, 2 L water: **2.85 mg/d**	Medium salt intake, 50% discretionary salt, 100% fluoridated salt, 2 L water: **3.31 mg/d**	High salt intake, 50% discretionary salt, 100% fluoridated salt, 1 L water: **3.53 mg/d**
Tea + water	2 cups of tea, 2 L water: **2.87 mg/d**	3 cups of tea, 2 L water: **3.34 mg/d**	4 cups of tea, 1 L water: **3.58 mg/d**
Tea + salt + water	2 cups of tea, high salt intake, 25% discretionary salt, 50% fluoridated salt, 1.5 L water: **3.21 mg/d**	2 cups of tea, high salt intake, 25% discretionary salt, 50% fluoridated salt, 2 L water: **3.33 mg/d**	4 cups of tea, low salt intake, 5% discretionary salt, 50% fluoridated salt, 1 L water: **3.63 mg/d**

Bold font was used to highlight the resulting exposure estimate

## Discussion

After analysis of the existing knowledge base, the Working Group of EFSA’s Scientific Committee came to the conclusion in their opinion that fluoride exposure during pregnancy may adversely affect the developing brain (EFSA Scientific Committee 2025). The Working Group identified unborn children as the most vulnerable population and considered the exposure of pregnant women towards fluoride to be critical for the risk assessment. Acknowledging existing uncertainties, the authors proposed a daily fluoride intake of 3.3 mg fluoride as the safe level. We note that the association between fluoride exposure and neurodevelopmental effects, specifically IQ reduction in children exposed in utero, is still being discussed in the scientific community. Several countries (for example France, Germany, Austria, and Switzerland) recommend adequate fluoride intakes for adults which are very similar to those recommended by EFSA in 2013, namely 2.9–3.5 mg per day, based on risk for dental fluorosis (DGE 2022; Della Bucher Torre and Jotterand Chaparro 2021; ANSES 2021). We therefore used the safe level for adults of 3.3 mg per person and day as a benchmark to be compared with individual exposures under realistic expsoure scenarios. The aim of our calculations was to explore how varying levels of exposure, mirroring possible individual behaviour, influences the cumulative fluoride exposure in the studied scenarios. We identified three main contributors for the daily cumulative fluoride exposure: (i) volume of black or green tea consumed, (ii) the use of fluoridated salt, and (iii) volumes of water intake. The exposure levels of these contributors were systematically varied in our analysis. The selected variations were derived from information we found in the literature or in the EFSA opinion (2025). Other contributors, even if they represent main contributors in individual cases, were held constant (fluoride exposure by food and by toothpaste), because suitable data was not available which could have been included in these scenarios. Although fluoridated toothpaste was included in all scenarios based on the EFSA value, we were unable to identify individualized usage data. Similarly, no data was available to estimate individual fluoride intake from food.

We have individualised the intake of tea, selecting black and green tea intake at several different levels. Fluoride exposure from eight cups of tea alone was already above the safe level and it could be easily seen that exposure from four cups of tea was so high that adding the additional fluoride intake by food and toothpaste would lead to exceedance of the safe level. Therefore, we calculated the cumulative exposure for exposures not higher than two cups of black or green tea. The contribution to exposure from herbal tea was very low and we refrained from calculating cumulative exposures including herbal tea (however, herbal tea is included as exposure source in the calculator tab provided with the supplemental Excel file). The observed difference in fluoride concentrations between herbal tea and black and green tea infusions might be due to the fact that herbal tea is grown in European countries with low fluoride content in the soil whereas black and green tea is cultivated in areas with high fluoride content in the soil.

While in the single contributor scenario of drinking eight cups of black or green tea daily (1200 mL/d) the safe level is exceeded, none of the scenarios with the different levels of salt intake per day, discretionary salt intake, and percentage of fluoridated salt leads to exceedance of the safe level. The same holds true for daily water intake.

In the cumulative scenarios, the cumulative exposure of modest daily salt intake and modest daily consumption of black or green tea is below the safe level also for high water consumption of 2 L/d. Otherwise, some of the constellations are leading to exceedance of the safe level.

In the scenarios without the use of fluoridated salt, consumption of up to two cups of black or green tea does not lead to exceedance of the safe level. In contrast, the safe level is exceeded in the scenarios without black or green tea consumption (*i.e.* for non-tea drinkers), if salt intake is high or when moderate (middle-range) intake is a accompanied by high percentages of discretionary salt and fluoridated salt use. For non-tea drinkers (black/green), who are not using fluoridated salt, the scenarios with the other parameters chosen by us do not lead to an exceedance of the safe level.

Of course, the choice of input parameters for fluoride intake from food can critically influence cumulative exposure. Some of the parameters selected by us are different from the parameters used by EFSA (2025). For example, we calculated our exposure scenarios with 310 mg fluoride/kg salt as it is used in Germany, whereas EFSA used 250 mg/kg. The data of salt consumption we used were from a German survey which provided the distribution of consumption in the childbearing age group. EFSA calculated the fluoride exposure by tea for the whole population, including also non-tea drinkers, whereas we performed our calculations for tea drinkers and non-tea drinkers separately. The exposure by food at the 95th percentile is calculated by EFSA to be 2.31 mg/d which is two times the exposure in our calculation. Adding the difference between 1.17 mg/d and 2.31 mg/d (1.16 mg/d) to the cumulative fluoride exposure would result in exceedance in nearly all of the scenarios.

We have not included additional sources of fluoride exposure in our scenarios, such as bottled natural mineral water and mouth wash, due to the lack of data on their usage patterns. Both mineral water and mouth wash may contribute to a relevant extent to the total exposure. Directive 2003/40/EC (European Commission 2003) limits the concentration of fluoride in bottled natural mineral water to 5 mg/L so that half a cup of mineral water could contain up to 0.375 mg of fluoride. Data from EFSA indicate that the upper bound level is 0.238 mg/L so that half a cup of mineral water would only contain 0.018 mg fluoride. Compared to the concentration of tap water of which the upper bound is 0.231 mg/L the difference is not relevant and because it can be assumed that mineral water is not consumed in addition to tap water but instead, the exposure towards fluoride would not increase to a relevant extent. The concentration of mouth wash is reported in the literature as 250–500 ppm (250–500 mg/L) (Reshetnyak et al. 2019). It is a reasonable assumption that 30 mL is used per occasion from which 1% may be swallowed. This would lead to an ingestion of 0.075–0.15 mg fluoride per use. Using these assumptions and further assuming twice daily use, the additional fluoride exposure from mouth wash could be up to 0.3 mg/d (in line with what EFSA calculated for toothpaste alone).

Generally, the exposure assessments of institutions which provide support for regulatory decisions have the aim to address the exposure of the whole population and are presented as statistical results (*e.g.* mean and 95th percentiles of the exposure, in mg/kg bw per day, in the population). In some instances, the exposure is calculated for specific subgroups, for instance age-related or gender-related. For example, EFSA in their opinion reported daily fluoride intake levels for children and adults ranging from 0.1 to 5.0 mg/d based on an analysis of several studies by EFSA’s NDA Panel. For the sub-population of pregnant women, EFSA collected data from several publications investigating fluoride exposure biomarkers, e.g. in spot-urine. Mean values for urinary fluoride concentration (as a surrogate for an exposure estimate) ranged from 0.44 mg/L in Canadian women and 3.58 mg/L in a group of Chinese women. In comparison, for a large cohort of ~ 1000 adults in Canada, a mean value of 0.94 mg/L is reported, while for several Chinese adult cohorts, mean values range from 0.36 mg/L to 3.62 mg/L (EFSA Scientific Committee 2025). Thus, considering mean biomarker concentration values on a population basis, no clear differences in fluoride exposure can be inferred for pregnant vs non-pregnant adults. But because the exposure is expressed in statistical terms and exposure may differ drastically between individuals (as is evidenced by the wide ranges of values reported for urinary biomarkers), the provided information does not inform individuals where they are placed in the exposure distribution and thus on their individual exposure. To enable individuals to know their fluoride exposure, an Excel spreadsheet is provided in the supplemental material that can be used to calculate individual exposure by selecting suitable exposure descriptors (*e.g.* one cup of black tea per day or high salt intake).

Overall, the only scenarios which would not lead to exceedance of safe fluoride intake levels, are the exposure scenarios without fluoridated salt and/or without black or green tea—even if the exposure by food at the 95th percentile is used. Hence, in the absence of knowledge on the consumption of fluoride by food and acknowledging the uncertainties associated with the risk for adverse developmental effects, in a precautionary approach pregnant women may consider to limit their consumption of black and green tea and to not use fluoridated salt. Of note, there are no labelling provisions regarding fluoride content in tea, while drinking water is strictly regulated, and the fluoride content in salt and dental care products is limited and appears on the label. We have shown that tea consumption can be a main contributor to total fluoride exposure in adults in Europe. Although it should be noted that occasional exceedance of the safe level as proposed by EFSA is unlikely to constitute a health risk, we see a need to limit fluoride content in tea and to provide information about fluoride content in tea products to consumers. This would enable risk prevention, especially for vulnerable populations.

## Supplementary Information

Below is the link to the electronic supplementary material.

Supplementary file1 (XLSX 29 KB)

Supplementary file2 (DOCX 32 KB)

## Footnotes

1 In this publication, mean describes the arithmetic mean.

2 This volume corresponds to one small pot as per ISO standard No. 3103:2019 “Tea — Preparation of liquor for use in sensory tests” (ISO 3103:2019).

3 namely Germany, Austria, Switzerland, Croatia, the Czech Republic, Denmark, Finland, France, Greece, Ireland, Italy, Lithuania, the Netherlands, Portugal, Slovakia, Sweden, and Spain.
