# Supplementary material for: Fluoride intake during pregnancy: calculation of realistic exposure scenarios for individual risk assessment
Source: Arch Toxicol. 2025 Aug 31;99(12):4931–40. doi: 10.1007/s00204-025-04143-8 (PMC12534287; doi:10.1007/s00204-025-04143-8)
Supplement: Supplementary file 2 — Supplementary file2 (DOCX 32 KB) [file 204_2025_4143_MOESM2_ESM.docx]

Supplemental Material

Table 1: Concentrations of fluoride in tea infusions found in the literature.

| **Tea bought in** | **Concentration (mg/L)** | **Reference** |
| --- | --- | --- |
| *Black and green tea* | | |
| Poland | 3.8 – 4.0 | (Jakubczyk et al. 2022) |
| Denmark | 0.3 – 4.5 | (Krishnankutty et al. 2022) |
| Slovenia | 0.6 -4.7 | (Pavlovič et al. 2023) |
| UK | 1.0 – 11.1 | (Ruxton and Bond 2015) |
| Poland | 0.8 – 6.0 | (Szmagara et al. 2022) |
| Ireland | 1.6 - 6.1 | (Waugh et al. 2016) |
| **Mean ± SD** | **3.13 ± 1.88** |  |
| *Herbal tea* | | |
| Poland | 0.04 – 0.09 | (Szmagara et al. 2022) |
| **Mean ± SD** | **0.056 ± 0.0018** |  |
